# Clinical outcomes and lung toxicities after lung SABR using dynamic conformal arc therapy: a single-institution cohort study

**DOI:** 10.1186/s13014-023-02227-2

**Published:** 2023-02-22

**Authors:** Emmanuel Mesny, Myriam Ayadi, Pauline Dupuis, Guillaume Beldjoudi, Ronan Tanguy, Isabelle Martel-Lafay

**Affiliations:** Service de Radiothérapie, Centre Léon Bérard, 28 Prom. Léa et Napoléon Bullukian, 69008 Lyon, France

**Keywords:** Lung SABR, DCAT, Radiation pneumonitis

## Abstract

**Background:**

Stereotactic ablative radiotherapy (SABR) is a validated treatment for early stage lung cancer and pulmonary metastases. It provides a high local control rate with low symptomatic toxicities. Recently, Dynamic Conformal Arc Therapy (DCAT), a delivery option that differs from conventional DCA, has been implemented in the Monaco Treatment Planning System for SABR. The aim of the study was to report clinical outcomes and toxicities for patients treated for lung SABR with this new technique.

**Methods:**

We retrospectively identified adult patients treated for primary or secondary lung tumors with DCAT-SABR and reported their clinical, radiological, histological characteristics and dosimetric parameters. Total dose was delivered in 3 or 5 fractions for 95% of patients and prescribed on the 80% isodose line to the PTV periphery.

**Results:**

145 patients met inclusion criteria for a total of 152 lesions with a median follow up of 12 months. Local control for the irradiated site was 96.7% at 1 year. Overall survival was 93.1% at 1 year. Mean prescription dose in BED_10_ was 110 Gy. 92% of patients had a prescribed dose superior to 100 Gy BED_10_. Mean PTV coverage was 95.1%. There were 66 cases of grade 1 radiation pneumonitis (RP) (43%) and only 7 cases of symptomatic grade 2 RP (4.6%).

**Conclusion:**

Lung SABR for primary or metastatic lung tumors using dynamic conformal arc therapy provides efficient results of local control and low lung toxicities, similar to other SABR techniques. Advances in knowledge: SABR using DCAT is a safe technique to treat lung lesions, allowing intra-fraction motion limitation, potentially higher OARs protection and a shortened beam delivery.

**Supplementary Information:**

The online version contains supplementary material available at 10.1186/s13014-023-02227-2.

## Introduction

Stereotactic ablative radiotherapy (SABR) is the standard treatment for medically inoperable early-stage non-small cell lung cancer (NSCLC) [1]. More recently, it has become an option for the treatment of recurrent lung cancer or pulmonary metastasis [2]. Lung SABR provides high local control rates, usually ≥ 90% [3]. Radiation pneumonitis (RP) is one of the most common toxicities occurring after lung SABR [4, 5]. Most of the time, RP is asymptomatic (grade 1, i.e. only radiographic images). Only a small part of patients (up to 10%) will develop a symptomatic RP (sRP) (i.e. ≥ grade 2) after SABR [6, 7].

Lung SABR can be achieved by several treatment strategies of variable complexity, implying different beam deliveries, tumor motion management, dose calculation algorithms [8]. In our institution, patients with early stage NSCLC were initially treated using an Internal Target Volume (ITV) strategy and 3D conformal radiotherapy (3D CRT) consisting in ten to twelve coplanar fixed beams. Dose calculation was performed with a type B algorithm, first with Superposition Convolution (XiO, CMS) and then with Collapsed Cone Convolution (Monaco, Elekta, Crawley UK). Recently, Dynamic Conformal Arc Therapy (DCAT) was implemented in the Monaco Treatment planning System (TPS) (version 5.0 51.10, Elekta, Crawley, UK). Contrary to conventional DCA, this delivery option allows variable gantry speed and dose rate, and produces a limited modulation across the Planning Target Volume (PTV) thanks to the Segment Shaped Optimization (SSO), thus protecting neighboring organs at risk (OARs) while keeping a reduced interplay effect. The Monaco DCAT requires an inverse planning where dose objectives are applied to target volumes and doses constraints to OARs. The dose calculation is performed with Monte Carlo (XVMC), a type C algorithm. The purpose of this study was to clinically evaluate the transition from 3D CRT to the new implemented DCAT by reporting clinical outcomes and toxicities of patients treated for lung SABR.

## Patients and methods

### Patient selection

We retrospectively identified adult patients treated for primary or secondary lung tumors with DCAT-SABR in our radiotherapy department between April 2019 and December 2020 and then reviewed their clinical, radiological, histological characteristics as well as dosimetric parameters. Patients were excluded if they were treated for two lesions within the same radiotherapy volume, had lung SABR for the primary lung lesion followed or preceded by a thoracic radiotherapy with conventional dose fractionation for the mediastinum, had an ultracentral lesion (≤ 1 cm from the proximal bronchial tree overlapping the trachea or main bronchi, the esophagus or the heart) or if there was a context of re-irradiation (field overlap ≥ 2 Gy).

### Radiotherapy planning

Patients had a four-dimensional computed tomography scan (4D-CT scan). An ITV was delineated either directly on the Maximum Intensity Projection Image (MIP) or was obtained from the union of the Gross Target Volumes (GTV) delineated on the extreme and intermediate phases of the 4D-CT scan, if the tumor was in the vicinity of the diaphragm. A 5 mm margin was added to ITV, in all directions, to obtain PTV according to the department guidelines.

DCAT treatment plans were performed on the average CT scan. They consisted in full or partial, single or two coplanar 6MV photons beam arcs. Calculations were completed in dose to medium using Monte Carlo algorithm with the following options: SSO, variable dose rate, a 2 mm dose grid resolution and a 1% statistical uncertainty per plan. Depending on the tumor location, tumor size and clinical considerations, total dose was delivered in 3 or 5 fractions in 95% of patients and prescribed on the 80% isodose line to the PTV periphery (except for one patient: prescription on the 95% isodose because of the proximity of the stomach). Peripheral tumors received 3 fractions whereas central tumors or tumors adjacent to the chest wall were treated in 5 fractions.

**Fig. 1:**
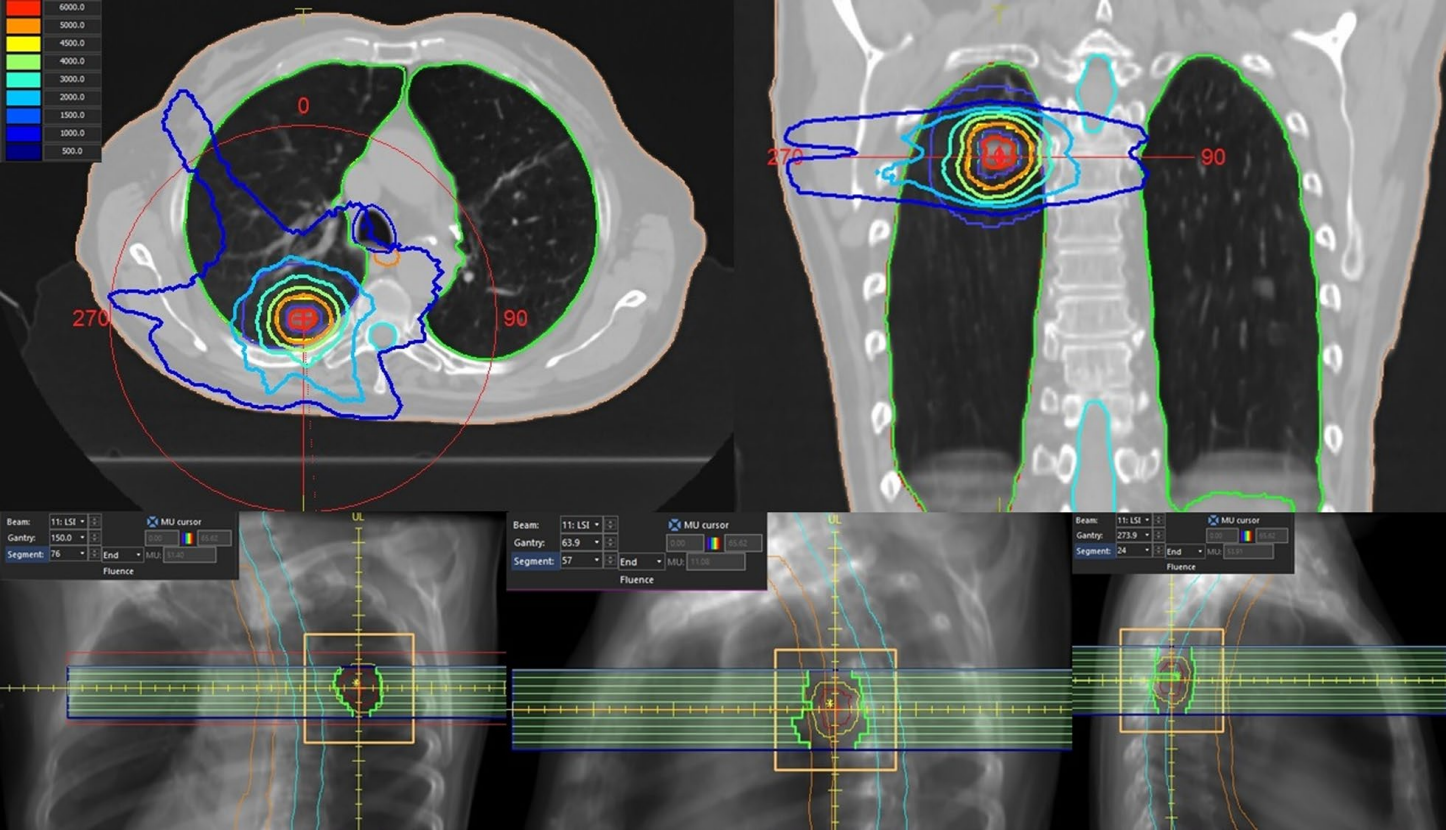
DCAT treatment of a tumor in the upper lung lobe from the patient cohort (50 Gy in 5 fractions): dose distribution and examples of segment shape of the beam delivery

As DCAT is based on inverse planning, dose objectives and constraints were applied on specific structures. For generating dose escalation into the tumor, a quadratic overdose and a maximum dose were especially applied to the ITV and the patient respectively, allowing then up to 125% of the total prescribed dose in the ITV (i.e. a prescription on the 80% isodose level). The dose conformity and the steep dose gradients were obtained using quadratic overdose constraints on the patient (shrink margins at several distances). A dose distribution example is provided on Fig. 1.

Treatments were delivered on a Versa HD (Elekta, Crawley UK).

### Data collection

Various dosimetric parameters were reported: dose fractionation, PTV volume, PTV coverage, dose received by 2% volume (D_2%_) of the PTV and the ITV, D_98%_ of the PTV and the ITV, median pulmonary density around the PTV (PDmed), conformity index (CI), Paddick’s Gradient Index (GI), biologically effective dose with alpha/beta of 10 (BED_10_), mean total lung dose (MLD_t_), mean ipsilateral lung dose (MLD_i_), V_t20_, V_t12.5_, V_t5_ of total lung-GTV, V_i20_, V_i12.5_, V_i5_ of ipsilateral lung-GTV, PTV maximum dose (Dmax), PTV and ITV median doses (Dmed) and PTV and ITV mean doses (Dmean). PDmed was estimated in a lung volume ring beyond 2 cm of the PTV. GI was the ratio of the isodose volume (IDV) of the 50% prescription dose to the 100% prescription IDV.

### Assessment of radiation pneumonitis

Patients were followed up clinically and radiologically (CT scan) every four to six months after completion of SABR treatment. Tumor response was assessed and RP was graded using CTCAE version 4.0 based on clinical and imaging data.

### Statistical analysis

Categorical comparisons were performed using Fisher’s exact test. Mann–Whitney test was used for quantitative variables. We computed Pearson *r* for correlation. Binary logistic regression was performed for univariate analysis to assess the relationship between risk factors and RP. The survival time was measured from the date of the beginning of the radiotherapy to the date of last follow-up or death. The probability of survival was estimated using the Kaplan–Meier method. All calculations were performed using SPSS software package version 28.0 (SPSS Inc, IBM Corp, Armonk, New York), and *P* values < 0.05 (two-sided) were considered significant. The design of the study was approved by the institutional review board and conducted according to European ethical guidelines.

## Results

We retrospectively identified 145 patients who met inclusion criteria for a total of 152 treated lesions (flowchart in Additional file 1 and patient’s characteristics are summarized in Tables 1 and 2). Seven patients had two distant pulmonary lesions treated sequentially. Median follow up was 12 months [1.0–26.5]. Local control (LC) for the irradiated site was 96.7% at 1 year (95% CI 94.0–99.3). Overall survival was 93.1% at 1 year (95% CI 89.1–97.3). Distant or loco-regional progression was observed in 53 patients (35%) during follow-up. Mean prescription dose in BED_10_ was 110 Gy; 8% of the patients had a prescription dose in BED_10_ inferior to 100 Gy (due to the proximity with an OAR or PTV size) and 66% had a BED_10_ prescription dose strictly equal to 100 Gy (i.e.: 50 Gy in 5 fractions). Mean PTV coverage was 95.1%. PTV coverage was < 90% for 11 patients (7.2%): 8 because of low PDmed (< 0.15) and 3 because of the proximity of OARs (heart). None of these 11 patients had a local failure at the time of the analysis. Median ITV D_mean_ was 59.8 Gy (BED_10_: 131.3 Gy), median PTV D_98%_ was 49.4 Gy (BED_10_: 98.2 Gy) and median PTV D_2%_ was 61.8 Gy (BED_10_: 138.2 Gy). Mean GI was 5.8 and was inversely correlated with the PTV size (*r* = −0.437 (p < 0.001), see Additional file 2).

**Table 1:** Baseline clinical and dosimetric characteristics (categorical variables)

Variable	Category	Number (%)
Sex	Male	108 (71%)
	Female	44 (29%)
Smoking history	Never smoker	33 (22%)
	Ex-smoker	99 (65%)
	Current smoker	20 (13%)
Performance status	PS < 2	132 (87%)
	PS ≥ 2	20 (13%)
History of COPD	No	67 (44%)
	Yes	85 (56%)
Confirmed diagnosis	Radiological confirmation	79 (52%)
	Histological proof	73 (48%)
Type of tumor	Primary tumor	127 (83%)
	Lung metastases	25 (16%)
History of lung surgery (lobectomy or pneumonectomy)	No	119 (78%)
	Yes	33 (22%)
History of CVD	No	96 (63%)
	Yes	56 (37%)
Diabetes	No	128 (84%)
	Yes	24 (16%)
Lung	Right	84 (55%)
	Left	68 (45%)
Tumor location	Lower lobe	70 (46%)
	Other lobe	82 (54%)
Tumor stage (8th edition)	T1a	26 (17%)
	T1b	84 (55%)
	T1c	24 (16%)
	T2a	9 (6%)
	T2b	7 (5%)
	T3	2 (1%)
Dose fractionation	5 × 10 Gy	100 (66%)
	3 × 18 Gy	35 (23%)
	5 × 9 Gy	5 (3%)
	3 × 16 Gy	3 (2%)
	10 × 5 Gy	6 (4%)
	8 × 7.5 Gy	1 (1%)
	3 × 12.5 Gy	1 (1%)
Abdominal compression	No	116 (76%)
	Yes	36 (24%)

*COPD* chronic obstructive pulmonary disease, *CVD* cardiovascular disease, *PS* performance status

**Table 2:** Baseline clinical and dosimetric characteristics (continuous variables)

Variables	Mean	Median	Range	SD
Age (y.o)	71.2	72.0	46–90	9.8
Smoking pack years	45.9	40.0	5–130	23.7
Tumor size (mm)	18.3	15.0	6.0–74	10.7
FEV1^a^ (%)	72.9	72.0	20–125	24.2
KCO^b^ (%)	71.0	68.5	15.0–118	23.3
ITV (cc)	9.5	4.6	0.5–115.3	14.8
PTV (cc)	25.7	17.8	2.20–167	24.4
PTV coverage (%)	95.1	96.4	71.5–99.7	4.7
PDmed	0.23	0.22	0.09–0.49	0.1
Conformity Index	0.81	0.79	0.54–0.93	0.1
Gradient index	5.9	5.57	3.43–11.18	1.72
ITV D_2%_ (Gy)	62.6	62.3	41.2–73.7	3.8
BED_10_ ITV D2% (Gy)	153.5	139.6	62.4–232.4	36.8
ITV D_98%_ (Gy)	56.2	56.2	38.5–63.5	3.4
BED_10_ ITV D_98%_ (Gy)	129.9	119.4	57–190.1	29.2
ITV Dmax (Gy)	64.0	63.6	42.2–75.8	3.9
BED_10_ ITV Dmax (Gy)	159.1	144.5	64.5–240.2	38.4
ITV Dmed (Gy)	60.0	59.9	39.8–69.9	3.5
BED_10_ ITV Dmed (Gy)	143.4	131.7	59.6–209.6	32.9
ITV Dmean (Gy)	59.8	59.8	39.8–69.7	3.6
BED_10_ ITV Dmean (Gy)	142.8	131.3	59.6–208.0	33.2
PTV D_2%_ (Gy)	62.0	61.8	41.2–73.2	3.7
BED_10_ PTV D_2%_ (Gy)	151.4	138.2	62.4–226.0	35.8
PTV D_98%_ (Gy)	49.5	49.4	36.0–58.6	3.2
BED_10_ PTV D_98%_ (Gy)	106.4	98.2	54.5–155.4	24.3
PTV Dmax (Gy)	64.0	63.6	43.0–75.8	3.8
BED_10_ PTV Dmax (Gy)	159.1	144.5	66.1–240.2	38.4
PTV Dmean (Gy)	56.2	56.2	39.3–65.7	3.0
BED_10_ PTV Dmean (Gy)	129.1	120.3	58.6–186.6	28.1
PTV Dmed (Gy)	56.2	56.4	39.4–66.5	3.0
BED_10_ PTV Dmed (Gy)	129.1	119.4	58.8–187.6	27.6
MLD_t_ (Gy)	3.4	3.1	0.10–19.9	1.9
V_t20_ (%)	3.9	3.5	0.80–10.3	2.1
V_t12.5_ (%)	7.1	6.7	1.7–18.6	3.4
V_t5_ (%)	15.9	14.7	3.5–36.5	6.7
MLD_i_ (Gy)	5.4	5.0	1.6–27.8	2.8
V_i20_ (%)	7.7	6.5	1.5–30.0	4.4
V_i12.5_ (%)	13.9	13.0	3.3–35.9	6.8
V_i5_ (%)	26.0	25.2	7.2–48.1	9.3

*FEV1* forced expiratory volume in one second, *KCO* diffusion capacity of the lung for carbon monoxide, *ITV* internal target volume, *PTV* planning target volume, *BED*_*10*_ biologically effective dose with alpha/beta of 10, *PDmed* median pulmonary density around the PTV, *LD* mean lung dose, *t* total (2 lungs), *i* ipsilateral lung, *Dmed* median dose, *Dmean* mean dose, *SD* standard deviation

^a^Data available in 113 patients

^b^Data available in 82 patients

RP occurred in 73/152 cases (48%). Median time for RP onset was 18 weeks [range 10–41]. There were 66 cases of grade 1 RP (43%) and only 7 cases of grade 2 sRP (4.6%). No ≥ grade 3 RP occurred in the entire cohort. None of the 7 cases of sRP had systemic treatment (immunotherapy or chemotherapy) within six months before or after lung SABR. The median tumor size of patients presenting sPR was 19.8 mm [range 10–45]. There were no death attributed to radiation pneumonitis. For clinical variables on univariate binary logistic regression analysis (see Additional file 3), history of lung surgery (Odds ratio (OR)-10.45, 95% CI 1.93–56.66) and age (OR 1.14, 95% CI 1.02–1.28) were significantly associated with sRP. sRP was not significantly associated with tumor size, tumor location or pulmonary function test. For dosimetric variables on univariate binary logistic regression analysis, V_i5_ (OR 1.12, 95% CI 1.03–1.23), V_i12.5_ (OR 1.11, 95% CI 1.01–1.22), V_t5_ (OR 1.12, 95% CI 1.01–1.24), V_t12.5_ (OR 1.29, 95% CI 1.06–1.56) were significantly associated with sRP. sRP was not significantly associated with PTV size, all BED_10_ ITV/PTV, GI or MLD.

## Discussion

In this retrospective series, we have reported the results of LC and pulmonary toxicity in a large cohort of patients treated for lung SABR by the newly implemented delivery technique DCAT. The average PTV coverage was 95.1% for all lesions. A PTV coverage of 95% is not an absolute prerequisite for the treatment plan validation if a minimum ITV_mean_ dose of 150 Gy BED_10_ is obtained [9]. In our study the dosimetric recommendation of ACROP was not fully achieved with a median ITV_mean_ dose of 131.3 Gy BED_10_ (< 150 Gy) and a median PTV _D98%_ of 98.2 Gy BED_10_ (< 100 Gy) but the median PTV D_2%_ was superior to 60 Gy. These results can be explained by the differences in the prescription dose with BED_10_ inferior to the recommended regimen of 15 Gy in 3 fractions (BED_10_: 112.5 Gy). However, it did not affect the LC rate which, in our study, is consistent with published rates, varying between 89 and 96% for primary lung tumors [10–12] and between 81 and 92.1% at one year for lung metastasis [2, 13, 14]. However, due to our short median follow-up we could not performed a robust long-term assessment of LC. A longer follow-up would have let to a more accurate recurrence rate, knowing that the majority of local recurrence occurs during the 5 years after SABR.

We reported an incidence of sPR of 4.6%. This is consistent with the incidence rates in the literature, ranging from 5 to 23% [6, 7, 15, 16]. Patients treated with surgery for lung cancer are at risk of second primary lung cancers. In this situation, lung SABR is often preferred because of a decreased lung volume. We found that an history of lung surgery was a significant risk factor for sPR. It may be explained by a reduced normal lung volume. In a recent systematic review of lung SABR after pneumonectomy, Arifin et al. reported a rate of grade 3 PR of 13.2% [17]. In these high-risk patient population, lung SABR should be done with caution. Although some studies suggest that PTV size and tumor location may influenced the risk of RP [6, 7], these variables were not found to be statistically associated with sPR in our analysis. In UK SABR cohort, where patients mostly received a three-fractions schedule, the median PTV was 30.3 cc and a PTV > 27.15 cc was significantly associated with the risk of PR [7]. Noticeably, the median PTV was smaller (median: 17.8 cc) in our cohort and the 5 fractions regimen was used most frequently.

One of the main objectives of this clinical retrospective study was to make sure that the planning optimization and delivery changes did not increase clinical toxicity. Many studies have demonstrated that algorithm performances in heterogeneities are not equal [18]. The ACROP guidelines for lung SABR recently reported that a type B algorithm was a mandatory requirement regarding the TPS specifications [8]. The transition from type B to type C algorithm improved the dose calculation accuracy [19, 20] but might have some clinical implications [21]. To evaluate the impact of such change, Frass et al. underlined the importance to correlate dosimetric variables to LC and patient toxicities from retrospective clinical studies [22]. Additionally, in lung SABR, especially when treating very small tumors surrounded by very low-density lung tissue, the loss of charged particle equilibrium combined to an increase of the penumbra width produces an underdosage of the tumor. If in the inverse planning the target objective of a minimum PTV coverage of 95% is fixed, the use of a Monte Carlo optimization could thus lead to an increase of segments size and/or a total MUs. The consequences of this phenomenon might be an increased normal lung tissue exposure. To the best of our knowledge this is the first clinical retrospective study assessing the LC and lung toxicity from patients treated with the DCAT delivery mode using Monte Carlo dose calculation.

Due to less monitor units (MU), DCAT can provide faster treatment delivery compared to other modalities (VMAT or Cyberknife) [23] and thus allows to decrease intrafraction uncertainties. In a recent study, MU for DCAT plans were found to be significantly lower by an average of 2.5 times as compared with VMAT plans for lung and liver SABR [24]. As expected, the GI found in our study is less steeper than those reported in literature with VMAT [25, 26]. Nevertheless we haven't found any significant correlation between PR and the GI. Thaper et al. performed a dosimetric comparison between VMAT and DCAT, using the Monaco TPS, for 25 patients treated with SABR for liver tumors [27]. Better conformity index and OARs sparing were found with VMAT plans. A higher degree of modulation obtained with VMAT plans resulted in a significant decrease of the dose spillage represented by the ratio of the 50% prescription isodose volume to the PTV volume (R50). Similarly, Stathakis et al. reported lower R50 with VMAT in 17 out of 19 patients [24].

Our study had several limitations. First, it is a retrospective and monocentric study, carrying all of the biases inherent to such an analysis, especially for the assessment of PR. Secondly, we have not compared DCAT with other lung SABR techniques (VMAT, multi beam IMRT, Cyberknife). Then, different protocols of dose prescription were used in the study making it difficult to analyze the dosimetric parameters. However, this study highlighted that DCAT was an appropriate and safe delivery option for peripheral or central lung tumors treated in free breathing with SABR.

## Conclusion

Lung SABR for primary or metastatic lung tumors using dynamic conformal arc therapy provides efficient results of local control and low lung toxicity rates. This technique, with a relatively short treating time, may be a reliable option for performing lung stereotactic irradiation.

## Supplementary Information

**Additional file 1.** Flowchart.
**Additional file 2.** Correlation between GI (on y-axis) and isodose volume of the 100% prescription dose (cc) (on x-axis).
**Additional file 3.** Univariate binary logistic regression analysis for the prediction of risk of sPR.

## Data Availability

The datasets used and/or analyzed during the current study are available from the corresponding author on reasonable request.

## References

[1] Vansteenkiste J, Crinò L, Dooms C, Douillard JY, Faivre-Finn C, Lim E, et al. 2nd ESMO consensus conference on lung cancer: early-stage non-small-cell lung cancer consensus on diagnosis, treatment and follow-up. Ann Oncol. 2014;25:1462–74. 10.1093/annonc/mdu089. 10.1093/annonc/mdu089 24562446

[2] Choi HS, Jeong BK, Kang KM, Jeong H, Song JH, Ha IB (2020). Tumor control and overall survival after stereotactic body radiotherapy for pulmonary oligometastases from colorectal cancer: a meta-analysis. Cancer Res Treat.

[3] Timmerman R, Paulus R, Galvin J, Michalski J, Straube W, Bradley J (2010). Stereotactic body radiation therapy for inoperable early stage lung cancer. JAMA.

[4] Barriger RB, Forquer JA, Brabham JG, Andolino DL, Shapiro RH, Henderson MA (2012). A dose-volume analysis of radiation pneumonitis in non-small cell lung cancer patients treated with stereotactic body radiation therapy. Int J Radiat Oncol Biol Phys.

[5] Kong F-MS, Moiseenko V, Zhao J, Milano MT, Li L, Rimner A, et al. Organs at risk considerations for thoracic stereotactic body radiation therapy: What is safe for lung parenchyma? Int J Radiat Oncol Biol Phys. 2021;110:172–87. 10.1016/j.ijrobp.2018.11.028. 10.1016/j.ijrobp.2018.11.028 PMC9454379 30496880

[6] Liu Y, Wang W, Shiue K, Yao H, Cerra-Franco A, Shapiro RH (2021). Risk factors for symptomatic radiation pneumonitis after stereotactic body radiation therapy (SBRT) in patients with non-small cell lung cancer. Radiother Oncol.

[7] Saha A, Beasley M, Hatton N, Dickinson P, Franks K, Clarke K (2021). Clinical and dosimetric predictors of radiation pneumonitis in early-stage lung cancer treated with Stereotactic Ablative radiotherapy (SABR)—an analysis of UK’s largest cohort of lung SABR patients. Radiother Oncol.

[8] Guckenberger M, Andratschke N, Dieckmann K, Hoogeman MS, Hoyer M, Hurkmans C (2017). ESTRO ACROP consensus guideline on implementation and practice of stereotactic body radiotherapy for peripherally located early stage non-small cell lung cancer. Radiother Oncol.

[9] de Jong EEC, Guckenberger M, Andratschke N, Dieckmann K, Hoogeman MS, Milder M (2020). Variation in current prescription practice of stereotactic body radiotherapy for peripherally located early stage non-small cell lung cancer: Recommendations for prescribing and recording according to the ACROP guideline and ICRU report 91. Radiother Oncol.

[10] Ball D, Mai GT, Vinod S, Babington S, Ruben J, Kron T, et al. Stereotactic ablative radiotherapy versus standard radiotherapy in stage 1 non-small-cell lung cancer (TROG 09.02 CHISEL): a phase 3, open-label, randomised controlled trial. Lancet Oncol. 2019;20:494–503. 10.1016/S1470-2045(18)30896-9. 10.1016/S1470-2045(18)30896-9 30770291

[11] Timmerman RD, Paulus R, Pass HI, Gore EM, Edelman MJ, Galvin J (2018). Stereotactic body radiation therapy for operable early-stage lung cancer: findings from the NRG oncology RTOG 0618 trial. JAMA Oncol.

[12] Shu Z, Dong B, Shi L, Shen W, Hang Q, Wang J (2020). Stereotactic body radiotherapy for elderly patients (≥ 75 years) with early-stage non-small cell lung cancer. J Cancer Res Clin Oncol.

[13] Yamamoto T, Niibe Y, Aoki M, Shintani T, Yamada K, Kobayashi M (2020). Analyses of the local control of pulmonary Oligometastases after stereotactic body radiotherapy and the impact of local control on survival. BMC Cancer.

[14] Klement RJ, Abbasi-Senger N, Adebahr S, Alheid H, Allgaeuer M, Becker G (2019). The impact of local control on overall survival after stereotactic body radiotherapy for liver and lung metastases from colorectal cancer: a combined analysis of 388 patients with 500 metastases. BMC Cancer.

[15] Takemoto S, Shibamoto Y, Hashizume C, Miyakawa A, Murai T, Yanagi T (2021). Changes in pulmonary function and their correlation with dose-volume parameters in patients undergoing stereotactic body radiotherapy for lung cancer. J Radiat Res.

[16] Zhao J, Yorke ED, Li L, Kavanagh BD, Li XA, Das S (2016). Simple factors associated with radiation-induced lung toxicity after stereotactic body radiation therapy of the thorax: a pooled analysis of 88 studies. Int J Radiat Oncol Biol Phys.

[17] Arifin AJ, Al-Shafa F, Chen H, Boldt RG, Warner A, Rodrigues GB, et al. Is lung stereotactic ablative radiotherapy safe after pneumonectomy? A systematic review. Transl Lung Cancer Res. 2020;9:348–53. 10.21037/tlcr.2020.01.18. 10.21037/tlcr.2020.01.18 PMC7225144 32420074

[18] Knöös T, Wieslander E, Cozzi L, Brink C, Fogliata A, Albers D (2006). Comparison of dose calculation algorithms for treatment planning in external photon beam therapy for clinical situations. Phys Med Biol.

[19] Fotina I, Winkler P, Künzler T, Reiterer J, Simmat I, Georg D (2009). Advanced kernel methods vs. Monte Carlo-based dose calculation for high energy photon beams. Radiother Oncol.

[20] Bosse C, Narayanasamy G, Saenz D, Myers P, Kirby N, Rasmussen K (2020). Dose calculation comparisons between three modern treatment planning systems. J Med Phys.

[21] Chetty IJ, Devpura S, Liu D, Chen D, Li H, Wen NW (2013). Correlation of dose computed using different algorithms with local control following stereotactic ablative radiotherapy (SABR)-based treatment of non-small-cell lung cancer. Radiother Oncol.

[22] Fraass BA, Smathers J, Deye J (2003). Summary and recommendations of a National Cancer Institute workshop on issues limiting the clinical use of Monte Carlo dose calculation algorithms for megavoltage external beam radiation therapy. Med Phys.

[23] Rauschenbach BM, Mackowiak L, Malhotra HK (2014). A dosimetric comparison of three-dimensional conformal radiotherapy, volumetric-modulated arc therapy, and dynamic conformal arc therapy in the treatment of non-small cell lung cancer using stereotactic body radiotherapy. J Appl Clin Med Phys.

[24] Stathakis S, Narayanasamy G, Licon AL, Myers P, Li Y, Crownover R (2019). A dosimetric comparison between volumetric-modulated arc therapy and dynamic conformal arc therapy in SBRT. J BUON.

[25] Kim S-T, An HJ, Kim J-I, Yoo J-R, Kim HJ, Park JM (2020). Non-coplanar VMAT plans for lung SABR to reduce dose to the heart: a planning study. Br J Radiol.

[26] Giglioli FR, Strigari L, Ragona R, Borzì GR, Cagni E, Carbonini C (2016). Lung stereotactic ablative body radiotherapy: a large scale multi-institutional planning comparison for interpreting results of multi-institutional studies. Phys Med.

[27] Thaper D, Kamal R, Singh G, Oinam AS, Yadav HP, Kumar R (2020). Dosimetric comparison of dynamic conformal arc integrated with segment shape optimization and variable dose rate versus volumetric modulated arc therapy for liver SBRT. Rep Pract Oncol Radiother.

