# Supplementary material for: Clinical outcomes and lung toxicities after lung SABR using dynamic conformal arc therapy: a single-institution cohort study
Source: Radiat Oncol. 2023 Feb 22;18:36. doi: 10.1186/s13014-023-02227-2 (PMC9948312; doi:10.1186/s13014-023-02227-2)
Supplement: Supplementary file 1 — Additional file 1. Flowchart. Additional file 2. Correlation between GI (on y-axis) and isodose volume of the 100% prescription dose (cc) (on x-axis). Additional file 3. Univariate binary logistic regression analysis for the prediction of risk of sPR. [file 13014_2023_2227_MOESM1_ESM.docx]

**Supplementary files:**

214 patients (≥18 years old) treated by DCAT for lung SABR between April 2019 and December 2020.

- 27 patients with context of re-irradiation with a field overlap ≥ 2 Gy in the new PTV
- 10 patients with ultracentral lung tumors
- 4 patients treated simultaneously for two lesions in one volume
- 19 patients with lung SABR for the primary lung lesion followed or preceded by a thoracic radiotherapy with conventional dose fractionation for the mediastinum
- 1 patient with treatment interruption due to a serious COVID infection

145 patients included and analysed in the study

*Additional file 1:* **Flowchart**

*Additional file 2* : **Correlation between GI (on y-axis) and isodose volume of the 100% prescription dose (cc) (on x-axis).**

| **Variable** | **OR** | **CI 95%** | **p-value** |
| --- | --- | --- | --- |
| Age | 1.14 | 1.02-1.28 | 0.022 |
| History of lung surgery | 10.45 | 1.93-56.66 | 0.007 |
| Tumor size | 1.02 | 0.97-1.08 | 0.395 |
| Tumor location (inferior as reference) | 0.32 | 0.06-1.73 | 0.188 |
| FEV1 | 1.02 | 0.98-1.05 | 0.320 |
| V_i5_ | 1.12 | 1.03-1.23 | 0.013 |
| V_i12.5_ | 1.11 | 1.01-1.22 | 0.029 |
| V_i20_ | 1.13 | 0.99-1.28 | 0.074 |
| V_t5_ | 1.12 | 1.01-1.24 | 0.026 |
| V_t12.5_ | 1.29 | 1.06-1.56 | 0.011 |
| V_t20_ | 1.27 | 0.91-1.46 | 0.132 |
| MLD_t_ | 1.15 | 0.91-1.46 | 0.232 |
| MLD_i_ | 1.13 | 0.96-1.33 | 0.130 |
| BED_10_ PTV Dmax | 1.02 | 0.99-1.03 | 0.098 |
| BED_10_ PTV Dmed | 1.02 | 0.99-1.04 | 0.114 |

*Additional file* **3: Univariate binary logistic regression analysis for the prediction of risk of sPR**

*FEV1 : forced expiratory volume in one second ; OR : odd ratio; CI : confidence interval; PTV : planning target volume; BED_10_ : biologically effective dose with alpha/beta of 10; MLD : mean lung dose; t : total (2 lungs); i : ipsilateral lung*
